# Endothelial response to blood-brain barrier disruption in the human brain

**DOI:** 10.1172/jci.insight.187328

**Published:** 2024-12-26

**Authors:** Andrew Gould, Yu Luan, Ye Hou, Farida V. Korobova, Li Chen, Victor A. Arrieta, Christina Amidei, Rachel Ward, Cristal Gomez, Brandyn Castro, Karl Habashy, Daniel Zhang, Mark Youngblood, Crismita Dmello, John Bebawy, Guillaume Bouchoux, Roger Stupp, Michael Canney, Feng Yue, M. Luisa Iruela-Arispe, Adam M. Sonabend

**Affiliations:** 1 Department of Neurological Surgery,; 2 Northwestern Medicine Malnati Brain Tumor Institute of the Lurie Comprehensive Cancer Center, and; 3 Department of Biochemistry and Molecular Genetics, Feinberg School of Medicine, Northwestern University, Chicago, Illinois, USA.; 4 Institute of Biomedicine, College of Life Sciences, Inner Mongolia University, Hohhot, China.; 5 Department of Cell and Developmental Biology, Feinberg School of Medicine, Northwestern University, Chicago, Illinois, USA.; 6 Department of Neurosurgery, University of Chicago, Chicago, Illinois, USA.; 7 Rush Medical College, Chicago, Illinois, USA.; 8 Department of Anesthesiology, Feinberg School of Medicine, Northwestern University, Chicago, Illinois, USA.; 9 Carthera, Lyon, France.; 10 Department of Neurology and; 11 Division of Hematology and Oncology, Department of Medicine, Feinberg School of Medicine, Northwestern University, Chicago, Illinois, USA.

**Keywords:** Neuroscience, Vascular biology, Endothelial cells

## Abstract

Cerebral endothelial cell (EC) injury and blood-brain barrier (BBB) permeability contribute to neuronal injury in acute neurological disease states. Preclinical experiments have used animal models to study this phenomenon, yet the response of human cerebral ECs to BBB disruption remains unclear. In our phase I clinical trial (ClinicalTrials.gov NCT04528680), we used low-intensity pulsed ultrasound with microbubbles (LIPU/MB) to induce transient BBB disruption of peritumoral brain in patients with recurrent glioblastoma. We found radiographic evidence that BBB integrity was mostly restored within 1 hour of this procedure. Using single-cell RNA sequencing and transmission electron microscopy, we analyzed the acute response of human brain ECs to ultrasound-mediated BBB disruption. Our analysis revealed distinct EC gene expression changes after LIPU/MB, particularly in genes related to neurovascular barrier function and structure, including changes to genes involved in the basement membrane, EC cytoskeleton, and junction complexes, as well as caveolar transcytosis and various solute transporters. Ultrastructural analysis showed that LIPU/MB led to a decrease in luminal caveolae, the emergence of cytoplasmic vacuoles, and the disruption of the basement membrane and tight junctions, among other things. These findings suggested that acute BBB disruption by LIPU/MB led to specific transcriptional and ultrastructural changes and could represent a conserved mechanism of BBB repair after neurovascular injury in humans.

## Abstract 2

Targeted ultrasound is used to study how human cerebral endothelial cells respond to acute blood-brain barrier disruption.

## Introduction

The blood-brain barrier (BBB) is a complex network of multiple cell types that lines the neurovasculature. It plays a crucial role in safeguarding the central nervous system (CNS) from harmful substances and maintaining an environment optimal for neuronal function (1, 2). The cerebral endothelial cells (ECs) are a key component of this barrier, forming a continuous layer that restricts transcellular and paracellular transport through specialized tight junction (TJ) complexes, characteristic suppression of transcytosis, a dense basement membrane, and selective membrane transport proteins for essential nutrients and metabolites (3–6).

Cerebral ECs are integral to CNS homeostasis. Loss of barrier integrity has been implicated as a secondary mechanism of neuronal injury in acute neurological disease states, ranging from traumatic brain injury (TBI) to ischemic stroke (1, 2, 7–10). Animal studies have utilized electron microscopy to examine how sudden BBB disruption results in ultrastructural changes to ECs at the cerebral microvasculature, including the breakdown of TJs, increased transcytosis, and the breakdown of the basement membrane. These structural changes are a part of the “barrier breakdown” that results in pathological neurovascular permeability and contributes to the cerebral edema that serves as a secondary mechanism of neuronal injury in these disease states (11–14).

While this pathological permeability might be reversible, little is known about the process of barrier repair and return to BBB homeostasis in humans. Animal models have shown that acute BBB disruption induces cerebral ECs to alter transcription of genes related to intercellular adhesion, cytoskeletal organization, and attachment to the extracellular matrix. This implies that ECs are sensitive to barrier compromise and likely play a role in the repair process that involves structural reorganization of their attachments to each other and the surrounding environment (15, 16). Studying how human cerebral ECs respond to acute BBB disruption could elucidate how barrier integrity is restored, how CNS homeostasis is regained, and how to mitigate permanent neurological injury in these disease states.

The use of low-intensity pulsed ultrasound with microbubbles (LIPU/MB) has emerged as a technique to enhance the brain concentrations of systemically administered drugs for the treatment of tumors and other CNS diseases (17–23). In previous reports, including ours, LIPU/MB via a skull-implantable ultrasound array (the SonoCloud-9 or SC9, Carthera) was used to induce temporary BBB disruption in patients with recurrent glioblastoma (GBM) (24, 25). This method has proven to be a safe, reproducible, and feasible means of enhancing concentrations of multiple drugs in the human brain (24, 26). Using contrast-enhanced magnetic resonance imaging (MRI), we showed that BBB opening within brain regions targeted by LIPU/MB (hereafter referred to as “sonication”) resolves rapidly, as permeability to gadolinium contrast was mostly reduced within an hour after this procedure (24, 25).

Having established the feasibility and kinetics of sonication-induced BBB opening, we leveraged LIPU/MB as a means of studying acute BBB disruption within the human brain in a controlled and consistent timeframe. Through our phase I clinical trial NCT04528680, we used intraoperative sonication to induce transient BBB disruption in patients undergoing resection of recurrent GBM (24). After opening the BBB, we sampled noneloquent sonicated peritumoral brain within minutes of the procedure (when the BBB was most permeable) and again at approximately 45–60 minutes afterward, along with nonsonicated control tissues. Using single-cell RNA sequencing (scRNA-Seq) and transmission electron microscopy (TEM), we then studied the effects of ultrasound-mediated BBB disruption on the transcriptome and ultrastructure of microvascular ECs in the human brain.

## Results

### Transcriptional response of human cerebral endothelium to ultrasound-mediated BBB disruption.

**Figure 1:**
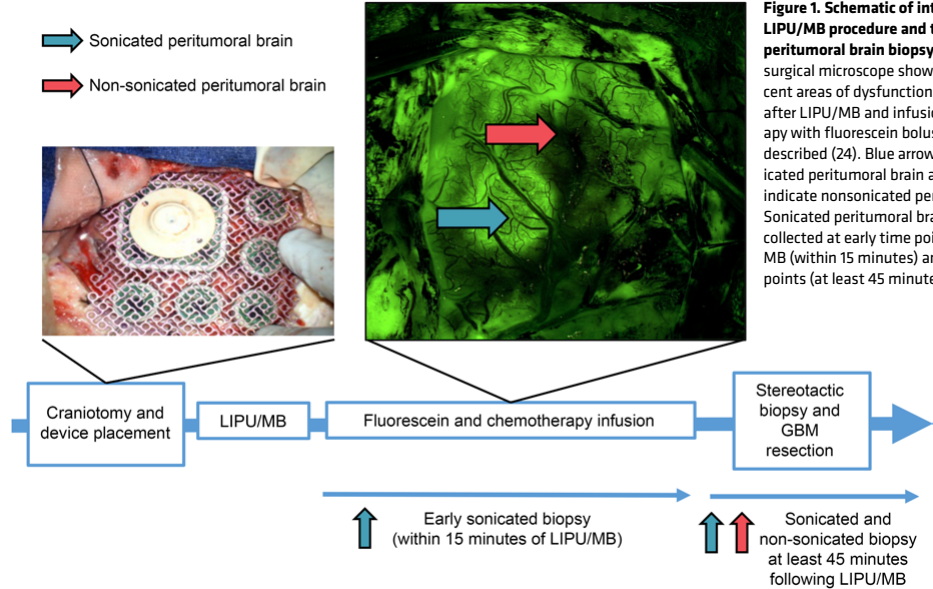
Schematic of intraoperative LIPU/MB procedure and time course of peritumoral brain biopsy. Photo from surgical microscope shows hyperfluorescent areas of dysfunctional BBB shortly after LIPU/MB and infusion of chemotherapy with fluorescein bolus as previously described (24). Blue arrows indicate sonicated peritumoral brain and pink arrows indicate nonsonicated peritumoral brain. Sonicated peritumoral brain biopsies were collected at early time points after LIPU/MB (within 15 minutes) and at later time points (at least 45 minutes after LIPU/MB).

We used scRNA-Seq to characterize the transcriptional response of human cerebral ECs to acute BBB disruption via sonication. As described previously, BBB disruption within sonicated peritumoral brain was mapped using fluorescein and fluorescence-based microsurgery (24). Fluorescein, which is typically restricted from crossing an intact BBB, accumulated in areas where the BBB was disrupted by LIPU/MB (22, 24). Thus, sonicated brain with increased BBB permeability exhibited notable fluorescence compared to adjacent nonsonicated brain not targeted by the ultrasound. A summary of the intraoperative LIPU/MB procedure and peritumoral biopsy process is shown in Figure 1.

**Figure 2:**
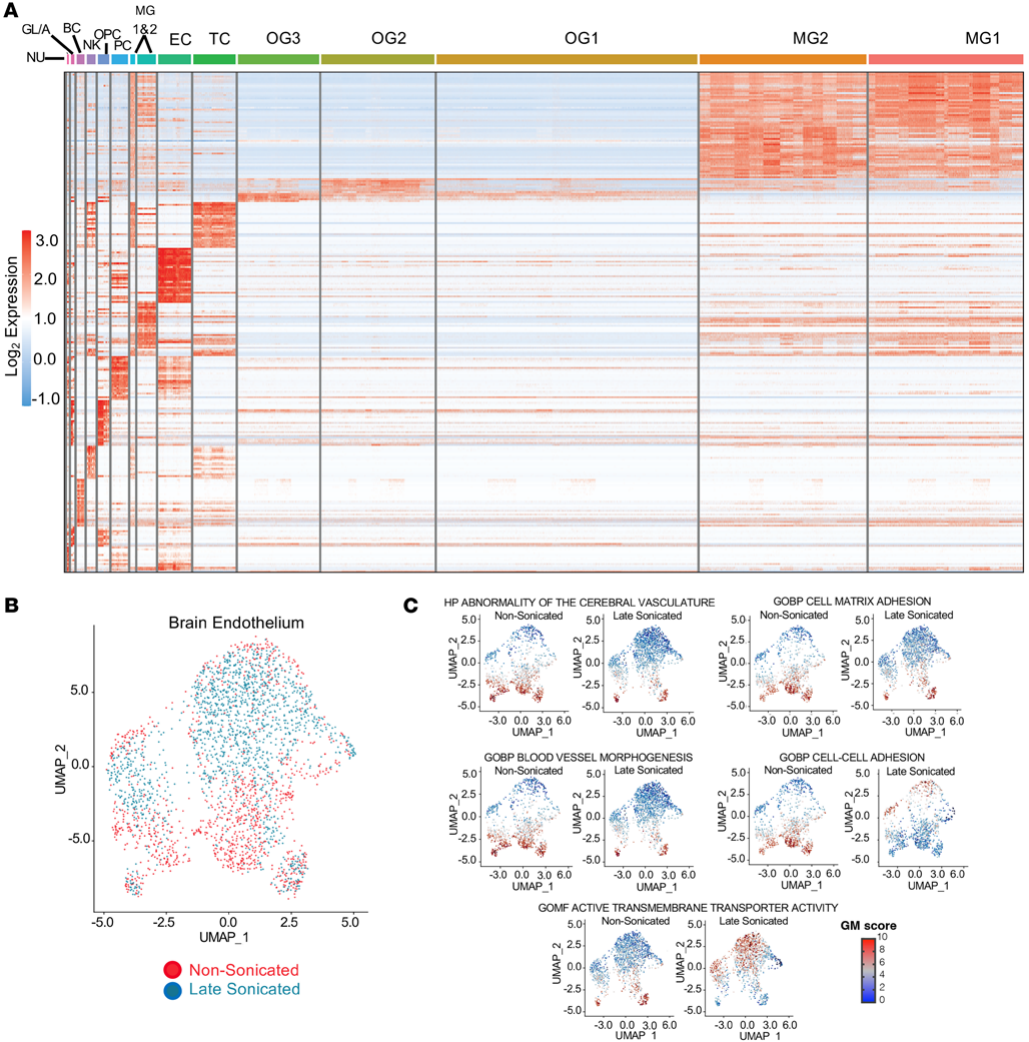
LIPU/MB-mediated BBB disruption alters the transcriptional phenotype of cerebral ECs. The single-cell transcriptional analysis derived from 6 patients with paired sonicated and nonsonicated peritumoral brain samples. Total of 2,643 ECs, including 1,470 from sonicated and 1,173 derived from nonsonicated peritumoral brain specimens collected approximately 45 minutes after LIPU/MB. (**A**) Heatmap illustrates the identity of 14 separate clusters of cells derived from the peritumoral brain, including ECs, isolated from sonicated and nonsonicated peritumoral brain tissues. Corresponding names for each cell type are listed along the top side of the heatmap (NU: neurons; GL/A: glioma cells and astrocytes; BC: B cell; NK: natural killer cell; OPC: oligodendrocyte progenitor cell; PC: pericytes; MC 1&2: monocytes 1 and 2; EC: endothelial cells; TC: T cells; OG1: oligodendrocyte 1; OG2: oligodendrocyte 2; OG3: oligodendrocyte 3; MG1&2: type 1 and 2 microglia). (**B**) Representative UMAP cluster showing gene expression profiles for ECs with sonicated subpopulation in blue and nonsonicated in red. (**C**) UMAP plots illustrate gene expression changes related to select GO themes that had significant differences between nonsonicated control and sonicated ECs (GSEA adjusted *P* < 0.05). HP, Human Phenotype; GOBP, Gene Ontology Biological Process; GOMF, Gene Ontology Molecular Function.

Each peritumoral brain sample was processed fresh into single-cell suspensions and subjected to scRNA-Seq library preparation (1 nonsonicated and 1 late sonicated peritumoral brain sample per patient, *N* = 6 patients, 12 brain samples in total). Unsupervised analysis led to 14 distinct gene expression–based cell clusters, designated as oligodendrocytes, microglia, T cells, ECs, monocytes, pericytes, oligodendroglial progenitor cells, natural killer cells, B cells, or glioma/astrocytes. We focused our analysis on ECs given their vital role in barrier function at the neurovasculature. We analyzed 2,643 ECs, including 1,470 from sonicated and 1,173 derived from nonsonicated peritumoral brain specimens (Figure 2A). A uniform manifold approximation and projection (UMAP) plot of these ECs was generated with cell labeling done according to whether these derived from late sonicated or nonsonicated control samples (Figure 2B).

**Figure 3:**
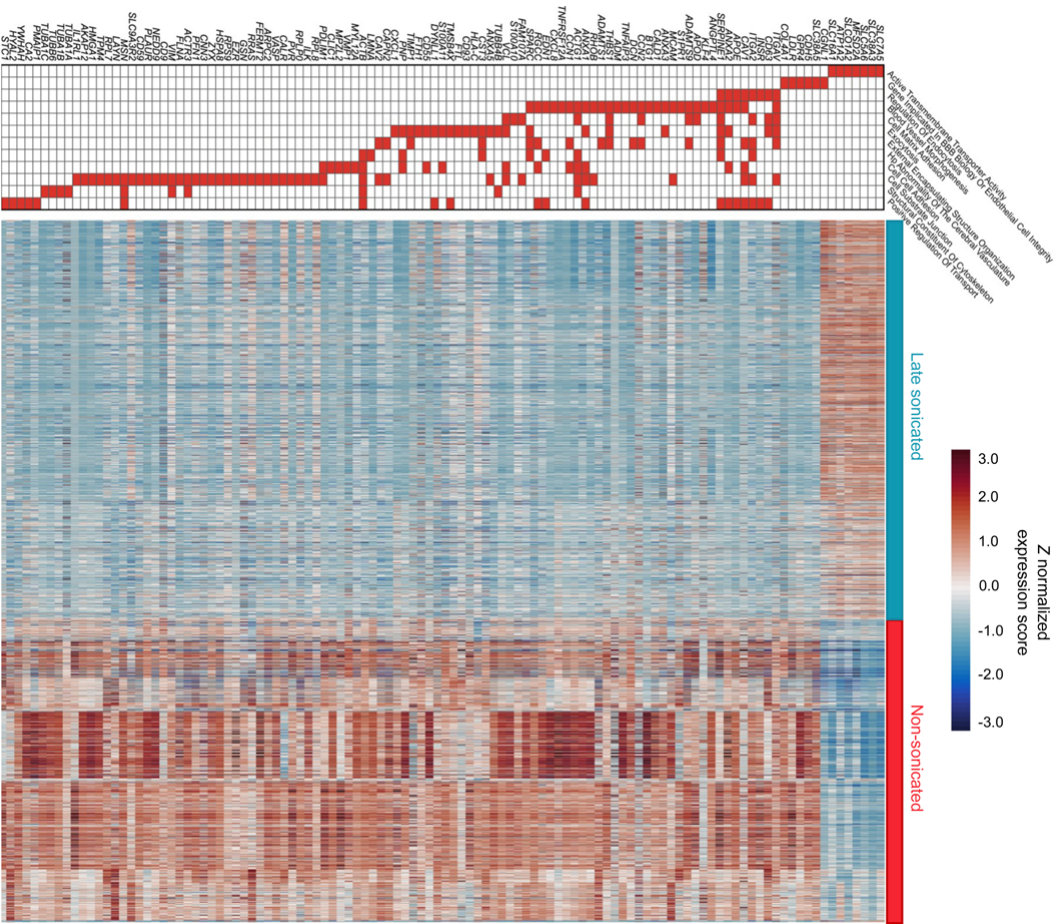
Gene expression changes in cerebral endothelial cells following LIPU/MB-mediated BBB disruption. Derived from 6 patients with paired sonicated and nonsonicated peritumoral brain samples. Heatmap illustrates *Z* score–normalized expression changes of individual genes altered between sonicated and control ECs from the GO themes described in Figure 2, as well as individual genes that have been implicated in BBB biology or EC homeostasis. The top of heatmap diagram shows the involvement of individual genes on each of these GO themes.

Gene set enrichment analysis (GSEA) of EC transcriptomics revealed significant alterations in gene transcription following sonication, impacting several ontology themes of interest in the context of the BBB (adjusted *P* < 0.05). Notably, there was downregulation of Gene Ontology (GO) themes Regulation of Endocytosis (normalized enrichment score [NES] = –2.01), Blood Vessel Morphogenesis (NES = –2.08), Cell Matrix Adhesion (NES = –2.13), Abnormality of Cerebral Vasculature (NES = –2.01), Structural Component of Cytoskeleton (NES = –1.96), and Cell-Cell Adhesion (NES = –2.04). Conversely, there was an upregulation of the theme Active Transmembrane Transporter activity (NES = 2.23) (Figure 2C). The heatmap in Figure 3 shows the expression changes of individual genes within these GO themes, comparing sonicated and nonsonicated brain ECs. Notable changes included altered transcription of genes previously implicated in neurovascular biology and barrier function. This included the downregulation of *GPR4* (log_2_ fold-change [log_2_FC] = –0.3748953, adjusted *P* = 1.08 × 10^–18^), a pH-sensing G protein–coupled receptor in cerebral ECs that modulates cAMP signaling and is crucial for cerebrovascular integrity (27, 28). Other alterations were in genes associated with selective transcytosis across the BBB. For example, we observed downregulation of transcripts for the gene of the LDL receptor (*LDLR*), expressed in cerebral ECs and used to mediate transcytosis (log_2_FC = –0.29, adjusted *P* = 1.61 × 10^–9^) (29, 30). Notably, sonication was also associated with significantly altered expression of various genes within the solute carrier (SLC) and organic ion (SLCO) superfamilies of membrane transport proteins. These transporters have been previously associated with influx and efflux of various substances across the neurovasculature and maintain a cerebral spinal fluid (CSF) ionic milieu that is conducive to proper neuronal development and function (31). Notable expression changes within these families included upregulation of genes *SLC38A3* (log_2_FC = 0.74, adjusted *P* = 1.14 × 10^–28^) and *SLC38A5* (log_2_FC = 0.28, adjusted *P* = 3.34 × 10^–4^), both coding for transporters specific for nitrogen-rich amino acids that can remove excess glutamine/glutamate from the CSF to the endothelium, likely as a means of avoiding excitotoxicity (31, 32); upregulation of *SLC7A5* (log_2_FC = 0.76, adjusted *P* = 9.47 × 10^–44^), a transporter of various neutral amino acids that also plays a role in glutamine/glutamate homeostasis in the CSF (31, 33); downregulation of *SLC4A7* (log_2_FC = –0.331, adjusted *P* = 4.78 × 10^–11^), a sodium/bicarbonate cotransporter responsible for maintaining appropriate ionic concentrations and pH in the CSF (34); and upregulation of *SLCO1A2* (log_2_FC = 0.553, adjusted *P* = 4.25 × 10^–15^) and downregulation of *SLCO4A1* (log_2_FC = –0.313, adjusted *P* = 2.64 × 10^–14^), both sodium-independent uptake transporters thought to play a role in drug delivery across the neurovasculature (35–37).

### BBB disruption alters EC and basement membrane morphology in brain capillaries.

Previous animal studies employed TEM to examine the effects of sonication on the ultrastructure of the cerebral microvasculature and ECs. These studies highlighted structural changes associated with acute BBB disruption, including irregular “opening” of the TJs between ECs that could facilitate paracellular drug delivery following sonication (38, 39). Therefore, we used TEM to study the ultrastructural changes induced by sonication to cerebral ECs in human peritumoral brain specimens. For this, we acquired peritumoral brain specimens from early-sonicated (within 15 minutes of LIPU/MB), late-sonicated (at least 45 minutes after LIPU/MB), and nonsonicated peritumoral brain biopsies (at least 45 minutes after LIPU/MB) from 3 separate patients (*N* = 9 tissue biopsies, 3 per patient). Using TEM, we then imaged capillary cross sections from each tissue specimen (nonsonicated *N* = 17, early sonicated *N* = 18, late sonicated *N* = 21) from each patient.

**Figure 4:**
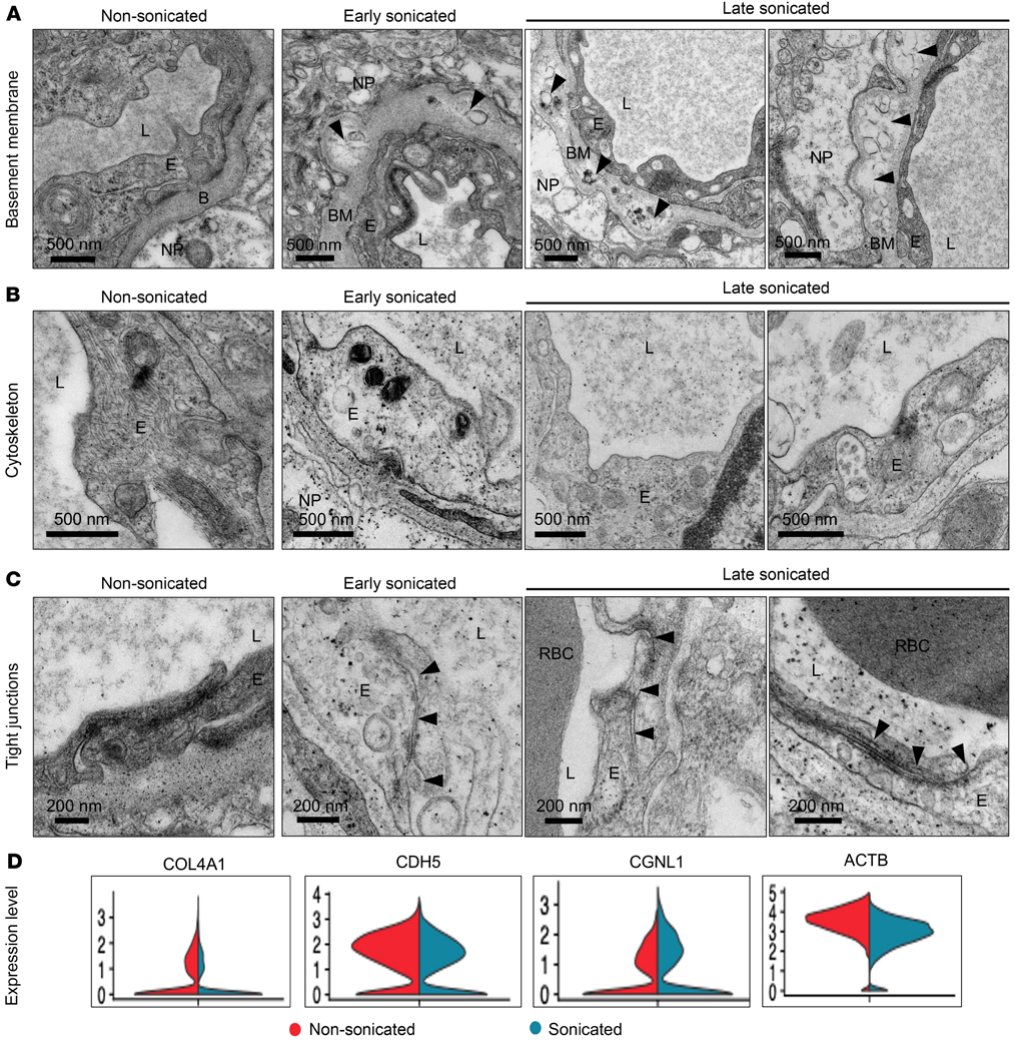
Ultrastructural alterations to brain capillaries following LIPU/MB-mediated BBB disruption. Representative electron micrographs highlighting various structural abnormalities observed in the cerebral capillaries of peritumoral tissues after sonication. Representative transmission electron micrographs with scale bars showing highlighted features from capillary cross sections acquired from nonsonicated peritumoral brain tissue, sonicated brain tissue early after LIPU/MB (4–15 minutes), and sonicated brain tissues late after LIPU/MB (57–63 minutes). Paired brain tissue samples from each time point were acquired from 3 separate patients, for a total of 9 tissue biopsies. Accompanying scale bars are used throughout, and relevant features of the capillary structure are denoted by the following letters: L (vascular lumen), E (endothelial cytoplasm), B (basement membrane), NP (neutrophil). (**A**) Sonicated capillaries frequently demonstrated irregular granular deposits or focal areas of amorphous enlargement, highlighted by arrowheads. (**B**) Within sonicated capillaries we also observed rarefaction of the endothelial cytosol and cytoskeletal disorganization. (**C**) We occasionally observed that TJ complexes appeared less dense than in their nonsonicated counterparts, with evidence of opening and irregular spacing between adjoining surfaces of the EC, with irregularities denoted by arrowheads. (**D**) Representative violin plots for normalized expression changes of genes that code for proteins associated with basement membrane (*COL4A1*), cytoskeleton (*ACTB*), or the TJ complex (*CDH5*, *CGNL1*) in sonicated (blue) and nonsonicated (red) tissues.

These electron micrographs were analyzed by an expert in cell biology and vascular pathology, who conducted a blinded review of the images. The expert was able to identify vessels as sonicated (either time point) and nonsonicated with 100% accuracy. A spectrum of key morphological distinctions was noted and used to distinguish sonicated and nonsonicated vessels. First, the basement membrane of sonicated capillaries frequently displayed granular or amorphous deposits that disrupted its continuity (Figure 4A). Second, sonicated ECs often showed evidence of cytosol rarefaction and disorganization of the cytoskeleton (Figure 4B). TJ complexes of sonicated ECs occasionally appeared less “dense” than their nonsonicated counterparts, sometimes with irregular spaces and “opening” of the intercellular cleft (Figure 4C). In line with these observations, our scRNA-Seq analysis revealed that sonication was associated with changes in the transcription of genes coding for structural components of the basement membrane and TJ/adherens junction complex. Notable changes included the downregulation of *COL4A1* coding for collagen type IV alpha 1 chain (log_2_FC = –0.34, adjusted *P* = 7.9 × 10^–10^), an essential component of the endothelial basement membrane linked to BBB integrity. In line with this, mutations in this gene have been implicated in intracerebral hemorrhage in mice (40, 41). We also noted downregulation of *CDH5*, which codes for cadherin-5 (log_2_FC = –0.288, adjusted *P* = 3 × 10^–7^), a major component of the adherens junctions found between cerebral ECs (1, 42). Conversely, there was upregulation of *CGNL1*, which codes for paracingulin, a protein localized to the cytoplasmic region of the apical portion of the TJ/adherens complex of brain ECs (log_2_FC = 0.457, adjusted *P* = 8.2 × 10^–3^) (6, 43, 44). We also observed downregulation of *ACTB* coding for β-actin (log_2_FC = –0.86, adjusted *P* = 2.8 × 10^–6^), a cytoskeletal protein whose remodeling has been implicated in reorganization of the endothelial TJs under periods of BBB permeability following mechanical stimuli and ischemic injury to the endothelium (Figure 4D) (5, 45). In sum, the combined results from our TEM and scRNA-Seq analyses indicate that LIPU/MB-induced BBB disruption is associated with marked changes to the morphology and transcriptional activity of human cerebral ECs that could be related to increased neurovascular permeability. These changes appear to exert a particularly strong effect on intercellular junctions, the basement membrane, and cytoskeleton.

### Ultrasound-mediated BBB disruption alters cerebral endothelial caveolar pit density in a time-dependent fashion.

**Figure 5:**
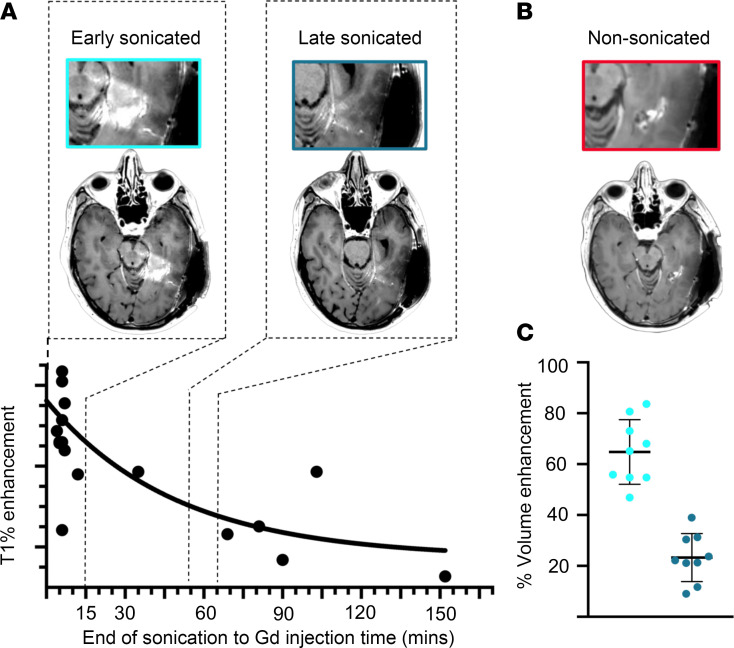
Radiographic estimate of BBB closure kinetics following acute disruption by LIPU/MB. (**A**) BBB permeability analysis from 17 patients, derived from post-LIPU/MB enhancement on MRI where variable time between LIPU/MB and gadolinium injection is shown on the *x* axis and percentage of T1 enhancement is on the *y* axis (derived from data reported previously) (24). Curve represents a linear mixed effects regression model (β = –0.2839; 95% CI –0.4254, –0.1424; *P* = 0.00056) as we previously reported (24). Above are corresponding T1 postcontrast MRI from a representative patient, showing the relative permeability of the BBB at the time point of gadolinium administration following LIPU/MB. The highlight outlined in light blue represents the relative permeability of the BBB shortly after sonication (within 15 minutes) and MRI highlighted in dark blue corresponds to permeability at least 45 minutes after LIPU/MB. Dotted lines drawn down from these highlights correspond to the time points of tissue acquisition presented on the *x* axis. (**B**) T1 postcontrast MRI, highlighted in red, from the same patient before sonication demonstrates the relative impermeability of nonsonicated brain. (**C**) Dot plots show change in percentage volume of enhancement in peritumoral brain targeted by ultrasound emitters from the SC9 device (*n* = 9 regions of peritumoral brain) when compared with baseline nonsonicated MRI in early sonicated (light blue) and late sonicated (darker blue). Data are shown as the mean ± SD. Figure generated from data previously reported (24).

**Figure 6:**
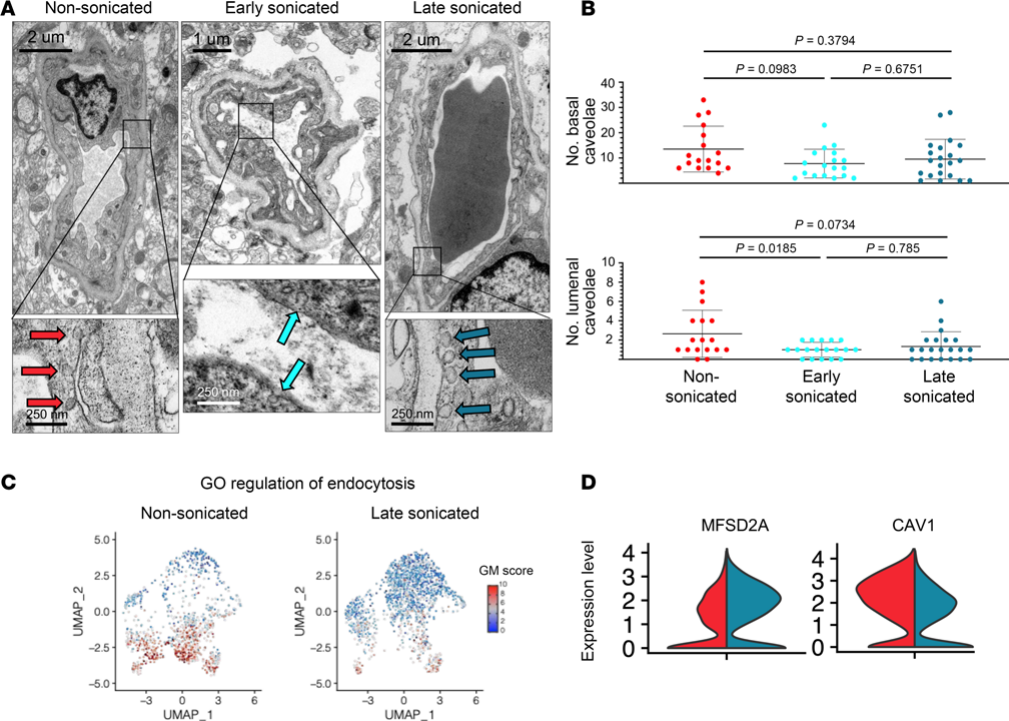
Ultrasound-mediated BBB disruption alters cerebral endothelial caveolar pit density in a time-dependent fashion. (**A**) Representative TEM micrographs with scale bars show capillary cross sections acquired from nonsonicated, early-sonicated, and late-sonicated peritumoral brain tissue. Permeability of the BBB at the time points of acquisition can be estimated by the radiographs in Figure 5A. Magnifications in the lower panels show caveolar pits attached to the basement and luminal membranes of the endothelium. Paired brain tissue samples from each time point were acquired from 3 separate patients, for a total of 9 tissue biopsies. Caveolae from early-sonicated brain are highlighted by light blue arrows, late-sonicated by darker blue, and nonsonicated by red. (**B**) Dot plots depict the total number of endothelial caveolae counted across all capillary cross sections at each time point (*N* for each group as follows: nonsonicated = 17, early sonicated = 18, late sonicated = 21), with colors matching the time points previously mentioned. Data are the mean ± SD. A mixed effects model was constructed considering sonication as a fixed effect and patient as a random effect influencing the number of caveolae. *P* values displayed on this panel are from a post hoc analysis and were obtained by likelihood ratio tests of the full model with the effect in question against the model without the effect in question. (**C**) UMAP plots demonstrate relative expression of genes pertinent to GO Theme Regulation of Endocytosis within nonsonicated and late-sonicated ECs. Legend for GM score on rightmost side. (**D**) Representative violin plots for normalized expression of genes *MFSD2A* and *CAV1* in both nonsonicated (red) and sonicated (blue) ECs from peritumoral brain tissues.

Building on previous animal studies that suggested that enhanced caveolar transcytosis in sonicated capillaries acted as a secondary mechanism of drug delivery across the BBB following sonication (39), we aimed to assess the density of endothelial caveolae in sonicated and nonsonicated peritumoral brain tissues. Since we previously found that peak BBB permeability after sonication occurred within 15 minutes of LIPU/MB (24), and barrier integrity returned quickly thereafter (Figure 5, A–C), we collected peritumoral brain specimens within this 15-minute window of maximum permeability. We counted well-formed caveolar pits (approximately 40–80 nm in diameter) that were attached to the basal and luminal membranes of the ECs (Figure 6A). Using a linear mixed effects model, we noted a significant effect of sonication on the frequency of luminal caveolar pits (χ^2^, *P* = 0.01542). Post hoc analysis showed decreased numbers of luminal caveolae in the peritumoral brain collected at the early-sonicated time point compared with the nonsonicated time point (χ^2^, *P* = 0.0185). Nonsignificant trends were found for numbers of luminal caveolae between late-sonicated and nonsonicated ECs (χ^2^, *P* = 0.0734). According to the same mixed effects model, sonication did not have an effect on the frequency of basal caveolae (χ^2^, *P* = 0.1049; Figure 6B). Post hoc analysis also showed no significant relationship between the frequency of basal caveolae counted in capillary cross sections, when comparing nonsonicated to early-sonicated (χ^2^, *P* = 0.0983), nonsonicated to late-sonicated (χ^2^, *P* = 0.3794), or early- to late-sonicated time points (χ^2^, *P* = 0.6751).

In line with these observations, our GSEA highlighted a downregulation in the GO theme Regulation of Endocytosis in sonicated ECs, as previously mentioned (NES = –2.01, adjusted *P* = 0.0432) (Figure 6C). We also noted that sonication altered the transcription of genes related to caveolar transcytosis, including increased expression of *MFSD2A* (log_2_FC = 0.592, adjusted *P* = 1.31 × 10^–6^) and decreased expression of *CAV1* (average log_2_FC in expression for sonicated ECs over nonsonicated cells = –0.77, adjusted *P* = 1.14 × 10^–32^) (Figure 6D). *MFSD2A* codes for a lysophosphatidylcholine symporter that has previously been characterized as essential to maintaining BBB function and repressing caveolar transcytosis at the cerebral endothelium, while *CAV1* codes for a protein component of caveolae (3, 4). Moreover, selective enrichment of Mfsd2a in rats was shown to attenuate caveolar transcytosis, BBB permeability, and neuronal injury in the days following experimentally induced subarachnoid hemorrhage (46, 47). Therefore, our TEM and transcriptional analyses could suggest that, within the 1-hour time frame we explored after BBB disruption, caveolar transcytosis does not appear to be enhanced in human cerebral ECs.

### BBB disruption by LIPU/MB leads to cytoplasmic vacuoles in ECs.

**Figure 7:**
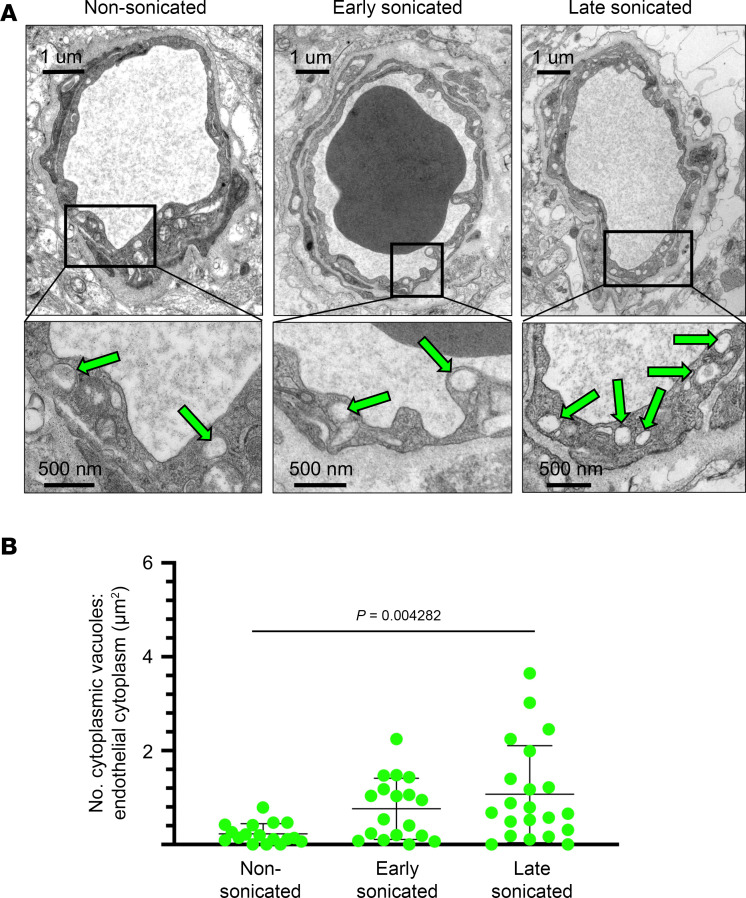
LIPU/MB-mediated BBB disruption increases endothelial cytoplasmic vacuoles over time. (**A**) Representative transmission electron micrographs with scale bars show capillary cross sections acquired from nonsonicated peritumoral brain tissue, sonicated brain tissue early after LIPU/MB (4–15 minutes), and sonicated brain tissues late after LIPU/MB (57–63 minutes). Paired brain tissue samples from each time point were acquired from 3 separate patients, for a total of 9 tissue biopsies. Magnifications in the lower panels show vacuoles within the endothelial cytoplasm, highlighted by green arrows. (**B**) Dot plots depicting the number of endothelial cytoplasmic vacuoles normalized to the total cross-sectional surface area of the total endothelial cytoplasm for each capillary (*N* for each group as follows: nonsonicated = 17, early sonicated = 18, late sonicated = 21). Data are the mean ± SD. *P* value is derived from a mixed effects model, constructed under the consideration of sonication as a fixed effect and patient as a random effect influencing the number of normalized vacuoles.

Upon further examination of our electron micrographs, we observed that sonicated ECs demonstrated large cytoplasmic vacuoles more frequently than nonsonicated ECs. These structures varied greatly in size but were much larger than and distinct from the membrane-bound caveolae noted previously (Figure 7A). To determine if these vacuoles were more frequent in sonicated blood vessels and to explore any time-dependent relationship to their frequency, we quantified their numbers in the EC cytoplasm. We then normalized these counts to the cross-sectional surface area of the EC cytoplasm in the micrograph for each vessel. Using a linear mixed effects model, we found that sonication had a significant effect on the frequency of these vacuoles (χ^2^, *P* = 0.004282; Figure 7B). The post hoc analysis highlighted a significant difference specifically between the late sonicated and nonsonicated groups (*P* = 0.0036). However, no significant differences were found for early-sonicated and late-sonicated groups (*P* = 0.3379) or early-sonicated and nonsonicated groups (*P* = 0.1313). These findings indicate that LIPU/MB-mediated BBB disruption leads to notable morphological changes within ECs, particularly in the formation of cytoplasmic vacuoles, that tend to increase over time.

## Discussion

**Figure 8:**
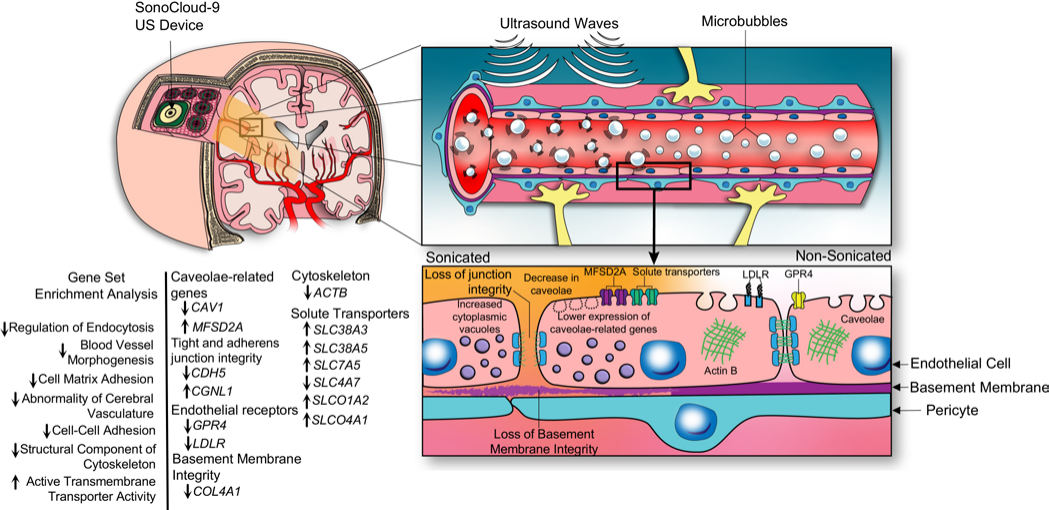
Illustration of transcriptional and structural consequences of ultrasound-mediated BBB disruption on the cerebral endothelium. Cartoon summarizing the key transcriptional and ultrastructural changes to the cerebral ECs that were observed following sonication.

Here we have leveraged scRNA-Seq and TEM to characterize the transcriptional response and ultrastructural changes of human cerebral ECs in an acute state of BBB disruption following LIPU/MB. Our study provides human data on the processes related to BBB disruption and restoration shortly after insult. A summary of some of the key structural and transcriptional changes is illustrated in Figure 8.

Previous studies characterized the transcriptome of the human ECs in health and vascular pathology (48–50). Yet, to our knowledge, transcriptional and structural changes in response to acute BBB disruption have been studied only in animal models. Many of the gene expression changes we identified, such as in *CDH5* and *COL4A1*, encode proteins that have previously been identified as structural components of the neurovascular unit and BBB, which impede passive diffusion of substances from the blood into the brain. Abnormal organization or absence of these components has been implicated in enhanced BBB permeability (40, 42). Other genes, such as *MFSD2A*, *CAV1*, *LDLR*, and *SLC/SLCO* family transporters, have previously been implicated in regulating transcytosis or allowing for selective delivery of substances across the BBB (3, 4, 30, 31).

Our GO analysis also revealed that sonication induced significant changes to themes related to intercellular and cell matrix adhesion, cytoskeletal organization, and vascular morphogenesis. Given that the established mechanism of LIPU/MB-enhanced drug delivery involves mechanical separation of ECs, these transcriptional changes could reflect a transient suppression of EC genes coding for components of the neurovascular ultrastructure, as suggested by our TEM analyses, wherein sonicated capillaries showed occasional disassembly of TJs, rarefaction of EC cytosol, and amorphous/granular deposits in the basement membrane that might reflect mechanical perturbation of the microvasculature. Some of these TEM findings have also been described in other preclinical models of BBB disruption, including LIPU/MB, ischemic stroke, and TBI (11, 14, 38, 51).

Using TEM, we observed a marked increase in the frequency of cytoplasmic vacuoles in sonicated ECs, which to our knowledge, has not been described previously. Their functional relevance remains unknown. Prior in vitro studies utilizing scanning electron microscopy noted that alternating acoustic pressures of ultrasound, with or without microbubbles, could form pores in cell membranes that render cells more permeable to drug delivery (52). The vacuoles we identified on TEM could be cross sections of these pores channeling through ECs, or alternatively, they could play some role in the pinocytosis of substances across the neurovasculature. However, we found these structures to be most frequent at a time point after sonication, when we observed permeability to gadolinium to already be greatly diminished (24). As permeability to gadolinium might differ to that of other substances, the potential contribution of these vacuoles to drug transport across the BBB remains to be determined.

With regard to transcytosis, our transcriptional analysis showed that sonication altered expression of various genes previously implicated in EC transporter activity and the regulation of endocytosis. This included increased expression of various *SLC/SLCO* family genes that are established regulators for concentrations of various metabolic substrates and ions in the brain interstitial space (31–37). It is possible that the increased expression of these transporters reflects a compensatory mechanism to correct abnormal concentrations of various amino acids and ions that could accumulate in the brain following sonication.

Contrary to preclinical TEM studies of LIPU/MB (38, 39), we did not find a time-dependent increase in the frequency of EC caveolae within an hour of sonication. However, we observed a time-dependent decrease in the frequency of luminal caveolar pits 4–15 minutes after sonication, a time point not explored by earlier studies (38). This discrepancy could have resulted from a difference in timing of tissue acquisition after sonication. Another possible explanation for this is that, while prior studies reported an increase in the number of EC caveolae, this effect was only statistically significant in arterioles (39). Given that our tissue biopsies were taken from the superficial cortex, most blood vessels we identified were capillaries with rare arterioles and venules. Thus, we restricted our analysis to capillaries and were unable to consider consequences of LIPU/MB on caveolar transcytosis at noncapillary components of the cerebral microvasculature. Another possibility could be that, in human cerebral ECs, caveolae do not play a substantial role in transcytosis following LIPU/MB. This is suggested by the increased transcription of the gene *MFSD2A*, which is known to inhibit caveolae-mediated transcytosis, as well as decreased transcription of *CAV1* that we noted in sonicated ECs. It has also been reported that caveolae have alternative functions unrelated to transcytosis, particularly at the neurovasculature. Prior electron microscopic studies performed in vitro reported that caveolae can “flatten” in response to mechanical forces such as uniaxial stretching. In this sense, they act as membrane redundancy or a “reservoir” that buffers mechanical stresses across the cell and protects it from rupturing (53). Moreover, integrin detachment and altered cell adhesion can cause caveolar pits to rapidly flatten and their density to decrease and then normalize within minutes upon readhesion (54). Consistent with this, we only encountered a decrease in caveolae in the luminal membrane of sonicated ECs, which are more likely to be directly targeted by the pressure of microbubble cavitation. Given that LIPU/MB is thought to mechanically separate adjacent ECs, the initial decrease in membrane-bound caveolae we observed immediately after sonication might reflect EC detachment from their intercellular connections and the underlying basal lamina. In line with this, normalization of the caveolar pit density within an hour of sonication coincided with partial restoration of BBB integrity (as also evidenced by our radiographic studies). Thus, our TEM analysis suggests that the immediate decrease in the frequency of caveolae could contribute to cellular resilience to mechanical stress, and BBB homeostasis following microbubble cavitation, while enhanced caveolar transcytosis may not contribute to increased permeability in human cerebral capillary ECs in a state of BBB disruption, at least within 1 hour of LIPU/MB.

Our study assessed the response of cerebral ECs to BBB disruption at a very acute time point (within an hour of sonication). We chose this time point for logistical reasons pertaining to chemotherapy infusion during the surgery, but it also coincided with our previous estimates on the kinetics of BBB and restoration of barrier function to gadolinium (24). Imaging studies in patients undergoing transcranial focused ultrasound have reported variable timelines on the return of barrier function. Some estimate persistent BBB permeability 2 to 6 hours after LIPU/MB, while others put it at 24 hours (21, 25, 55–57). This variability could be accounted for by differences in the acoustic parameters and the modality of ultrasound used to open the BBB in each study (transcranial versus skull implantable). Our study utilized a mechanical index of 1.03 MPa. This parameter was decided upon after previous clinical trials found it to be optimal for safe and effective BBB disruption using the SC9, where sound waves do not penetrate across bone (18, 56). In a recent publication by Carpentier et al., wherein patients with recurrent GBM underwent serial sonication-enhanced chemotherapy with the SC9, they reported hypointense lesions in susceptibility weighted imaging sequences seen on MRI suggestive of microhemorrhages in 6 out of 52 sonications (11%) (25).

While we focused on the response of the ECs to LIPU/MB, it is possible that the transcriptional and structural changes we report are not unique to LIPU/MB-based BBB disruption. Munji et al. examined the transcriptional alterations within the cerebral ECs of rodents in various experimental models of acute BBB disruption, including TBI, ischemic stroke, seizure, and autoimmune encephalomyelitis (15). Distinct transcriptional alterations were noted for each model, but there was also a core module of 54 genes whose transcripts were consistently enriched across all models at the time of peak BBB disruption. The authors speculated that this core module could reflect a conserved mechanism of regulating EC permeability and BBB repair (15). Some of these alterations were found on GO themes similar to those of our analysis, including cell adhesion–ECM receptor interaction and regulation of angiogenesis.

In conclusion, we characterized the transcriptional and ultrastructural alterations of LIPU/MB-mediated BBB disruption on human cerebral ECs at an acute time point. For this, we relied on intraoperative LIPU/MB of peritumoral brain to model this process in human cerebral tissues. We show that loss of BBB integrity is associated with altered expression of genes that relate to EC structure, attachment, and transcytosis. We also show that sonication alters the physical phenotype of ECs and the broader neurovascular ultrastructure. While our findings highlight acute changes seen after sonication, they present some similarity to EC changes reported in acute neurological disease states, where permeability of the BBB has been implicated; thus, our data might provide insight into mechanisms of BBB homeostasis and EC response to microvascular injury in the human brain seen in various neurological pathologies. Work should also be done to further characterize changes to cerebral ECs at later time points than ones we were able to explore. Though this presents obvious logistical hurdles, exploring the mechanisms of neurovascular permeability and recovery in the late stages of these diseases could reveal valuable targets for molecular therapies that may be used in the acute setting to attenuate permanent neuronal injury secondary to pathological BBB permeability.

## Methods

### Sex as a biological variable

Sex was not considered as a biological variable for the purposes of this study, due to availability of tissue samples. Tissues for this study were acquired from both male and female participants. The sex of each study participant is in Supplemental Table 1; supplemental material available online with this article; https://doi.org/10.1172/jci.insight.187328DS1

### Intraoperative LIPU/MB-enhanced chemotherapy and stereotactic biopsy of sonicated peritumoral brain

Enrolled patients received treatment as described previously (24). In brief, the use of intraoperative corticosteroids or mannitol was avoided for all cases where we performed intraoperative pharmacokinetics studies. Biopsy of noneloquent peritumoral brain was performed when feasible and justified as per standard neurosurgical technique. For these studies, we decreased the fraction of inspired oxygen as much as tolerated up to 20%, aiming to obtain an arterial O_2_ pressure < 100 mm/Hg, to model the outpatient setting where patients are on room air. We exposed the peritumoral brain to be excised, positioned the SC9 device in the cranial window, flooded the field with sterile saline, connected the device to the SC9 radiofrequency generator, and infused intravenous (IV) DEFINITY 10 μL/kg (Lantheus) microbubbles while sonicating the brain for a duration of 270 seconds using an acoustic pressure of 1.03 MPa, as was used in our recent clinical trials with the SC9 system (24, 25). Immediately after sonication, we infused fluorescein 500 mg IV and initiated a 45-minute IV infusion of nab-paclitaxel chemotherapy (Abraxane). LIPU/MB-based BBB opening was visualized and mapped using fluorescence microscopy (ZEISS Yellow 560 nm filter). Sonicated peritumoral brain was identified by fluorescence microscopy following infusion of fluorescein and nonsonicated peritumoral brain based on absence of fluorescence in this setting. Within 4–15 minutes of sonication (referred to as early time point), we obtained biopsies of ineloquent sonicated peritumoral brain where feasible, which were immediately fixed for TEM. Following the remainder of the 45-minute infusion period, we further biopsied paired sonicated and nonsonicated ineloquent peritumoral brain for additional TEM analysis and scRNA-Seq. Samples intended for sequencing were transported in saline on ice and underwent immediate processing. Representative fluorescence photographs of the brain and corresponding stereotaxic coordinates were obtained for each biopsy. This was followed by standard tumor resection and permanent implantation of the SC9 at the end of the procedure.

### scRNA-Seq

Patients whose tissues were used for scRNA-Seq analysis did not receive dexamethasone prior to obtaining these biopsies. RNA-Seq was performed for paired sonicated and nonsonicated peritumoral brain specimens collected at approximately 45 minutes after LIPU/MB per patient. Peritumoral brain was defined as brain parenchyma that was not enhancing per the contrast MRI used for stereotaxic navigation. Sonicated brain was identified by fluorescence microscopy following infusion of fluorescein and nonsonicated brain based on absence of fluorescence in this setting. Each tissue sample was processed fresh into single-cell suspensions and subjected to scRNA-Seq library preparation. Samples were transported on ice, and single-cell suspension was performed using the Miltenyi Biotec system on gentleMACS Octo Dissociator ]according to the manufacturer’s instructions. Isolated cells were washed with PBS containing 0.04% bovine serum albumin and filtered through a 40 μm cell strainer (MilliporeSigma). Cell concentration and viability were determined by a Countess II Automated Cell Counter (Thermo Fisher Scientific) with a final cell concentration of 700–1,200 cells/μL. scRNA-Seq libraries were generated using the Chromium Single Cell 3′ Reagent Kit (10x Genomics). Single-cell suspension was mixed with RT-PCR master mix and loaded together with Single Cell 3′ Gel Beads and Partitioning Oil into a Single Cell 3′ Chip (10x Genomics). The cDNA was amplified and further used to construct a 3′ gene expression library according to the manufacturer’s instructions. The size profiles of preamplified cDNA and sequencing libraries were examined by the Agilent High Sensitivity 2100 Systems. The scRNA-Seq library was sequenced on the Illumina NextSeq 500/550 platform.

### Single-cell transcriptomic analysis

All the scRNA-Seq data were aligned to GRCh38 reference genome and quantified using 10x Genomics Cell Ranger pipeline by running cellranger count. We kept the filtered data from Cell Ranger for further quality control (qc).

#### Doublet removal and qc.

The filtered_feature_bc_matrix generated by Cell Ranger pipeline was processed with Seurat (58). Cells with fewer than 200 unique genes or greater than 4,000 genes were removed. The remaining cells in each sample were used as the input of DoubletFinder (59). The first 20 principal components (PCs) with the proportion of artificial doublets = 0.25 and proportion of nearest neighbors = 0.09 were used to identify the doublets. The cells that were classified as doublets were then removed. The remaining cells from 12 samples were merged as a single Seurat object. To further remove the dead or dying cells, we filtered the cells by percentage of mitochondrial reads per cell greater than 15% or with greater than 20,000 counts.

#### Batch effect removal, dimensionality reduction, clustering, and cell annotation.

Cells from the Seurat object were analyzed with the standard workflow of Seurat. First, NormalizeData was run using the LogNormalize method and the scale factor with 10,000 for cell level normalization. The variable features were identified by findVariableFeatures using vst method with 2,000 features. The data were scaled to 10,000 unique molecular identifiers per cell and PCs were computed with RunPCA. The batch effect correction was performed using Harmony (60). UMAP was generated from the results of batch-corrected PCs. The cells were then clustered using FindNeighbors with batch corrected to 20 dimensions and FindClusters with a resolution of 0.5. Briefly, we determined the k-nearest neighbors of each cell and used k-nearest neighbors graphs to construct the shared nearest neighbors graph by calculating the neighborhood overlap (Jaccard index) between every cell and its k.param nearest neighbors to determine the unsupervised cell clusters. Cluster-specific marker genes were defined by Wilcoxon’s test with adjusted *P* < 0.01 and average logFC > 0.5. Clusters were annotated to cell types by comparing marker genes for each cluster to cell type markers from Panglaodb marker gene database (61) corresponding to expected human brain cell types. For example, *P2RY12* and *PTGS1* were used to define microglia cells; *CNP* and *PLP1* were used to define oligo cells; and *FTL1*, *LYZ*, and *IL7R* were used to define endothelial, monocyte, and T cell, respectively (48, 62–65).

#### Differential expression and functional enrichment analysis.

We performed differential expression analysis between sonicated and nonsonicated samples across each cell type using Wilcoxon’s test, and Benjamini-Hochberg method was used to estimate the FDR, following the recommendation of Seurat. The differentially expressed genes (DEGs) were filtered using average logFC > 0.5 and adjusted *P* < 0.05. The functional enrichment analysis for DEGs between sonicated and nonsonicated samples was conducted using clusterProfiler R package (66).

### Electron microscopy analysis

Patients whose tissues were used for ultrastructural analysis of peritumoral brain by TEM did not receive dexamethasone prior to obtaining the biopsies. For electron microscopy, approximately 1–2 mm^3^ samples of brain tissue, subjected to LIPU/MB or not, were excised and fixed in a mixture of 2.5% glutaraldehyde and 2% paraformaldehyde in 0.1 M cacodylate buffer for 2 or 3 hours or overnight at 4°C. After fixation, tissue was exposed to 1% osmium tetroxide and 3% uranyl acetate, dehydrated in ethanol, embedded in Epon resin, and polymerized for 48 hours at 60°C. Then ultrathin sections were made using Ultracut UC7 Ultramicrotome (Leica Microsystems) and contrasted with 3% uranyl acetate and Reynolds’s lead citrate. Samples were imaged using an FEI Company Tecnai Spirit G2 transmission electron microscope operated at 80 kV. Images were captured by Eagle 4k HR 200 kV charge-coupled device camera.

Caveolar pits were identified as membrane-bound invaginations (40–80 nm in diameter) that were directly attached to the basal and luminal surfaces of ECs. Caveolae were also distinguished from clathrin-coated vesicles according to their size, as well as by the density and absence of obvious protein spike along their membrane surfaces. Only well-formed caveolae, showing direct attachment to either of the endothelial membranes, were counted for this analysis. Cytoplasmic vacuoles were identified as single membrane vesicles ranging in sizes (150–250 nm in diameter) without any electron-dense content in majority cases.

### Statistics

We utilized a mixed effects linear model to assess the effect of sonication status on the frequency of basal and luminal endothelial caveolae and vacuoles relative to the cross-sectional surface area of the endothelial cytoplasm. For each type of cellular structure, models were constructed to compare a null scenario, considering interpatient variability, with an alternative model that included sonication status as a fixed effect. *P* values were obtained by likelihood ratio tests of the full model with the effect in question against the model without the effect in question. *P* < 0.05 was considered statistically significant. Post hoc analyses were also used to determine any relationship between the frequency of these structures at nonsonicated, early sonicated, and late sonicated time points.

### Study approval

This study was approved by the institutional review board of Northwestern University Feinberg School of Medicine (STU00212298), and all patients provided written informed consent, which included consent for the translational pharmacokinetics study and for nonidentifiable data collected to be included in scientific publications. Quality assurance monitors from the Clinical Trials Office at the Robert H. Lurie Comprehensive Cancer Center of Northwestern University verified the underlying study data and confirmed the accuracy of the results presented in this article.

### Data availability

The scRNA-Seq data have been deposited to the National Center for Biotechnology Information Gene Expression Omnibus (GSE208074). Supporting data values for all figures and analyses can be found in the Supporting Data Values file, which can be found in the online supplemental material.

## Author contributions

Single-cell suspension was performed by LC, CD, VAA, and BC. scRNA-Seq analysis was performed by YL, YH, and MY under the supervision of FY. Electron microscopy and related analyses were performed by FVK, AG, DZ, and MLIA. CA, RW, CG, JB, RS, and AMS managed the clinical and regulatory aspects of the clinical trial for the correlatives presented. GB and MC performed the imaging analysis and sonication-related technical assistance. The manuscript was drafted by AG, KH, VAA, and AMS. Statistical analysis was performed by VAA. Surgery and intraoperative LIPU/MB were performed by AMS with assistance from CA, CG, RW, AG, and JB. AMS designed and supervised the project.

## Supplementary Material

Supplemental data

Supporting data values

## Version 1 12/26/2024

In-Press Preview

## Version 2 02/24/2025

Electronic publication
