# Supplementary material for: Endothelial response to blood-brain barrier disruption in the human brain
Source: JCI Insight. 2024 Dec 26;10(4):e187328. doi: 10.1172/jci.insight.187328 (PMC11949064; doi:10.1172/jci.insight.187328)
Supplement: Supplemental data [file jciinsight-10-187328-s274.pdf]

Sex as Biological Variable

Supplementary Table 1 –
Sex As a Biological Variable

Single Cell Analysis Patient ID	Sex
1	Male
2	Female
3	Male
4	Male
5	Female
6	Female

Electron Microscopy Analysis Patient ID	Sex
7	Female
6	Female
8	Female
